# Performance Improvement for Detecting Brain Function Using fNIRS: A Multi-Distance Probe Configuration With PPL Method

**DOI:** 10.3389/fnhum.2020.569508

**Published:** 2020-11-06

**Authors:** Xinrui Chen, Xizi Song, Long Chen, Xingwei An, Dong Ming

**Affiliations:** ^1^Academy of Medical Engineering and Translation Medicine, Tianjin University, Tianjin, China; ^2^College of Precision Instruments and Optoelectronics Engineering, Tianjin University, Tianjin, China

**Keywords:** functional near-infrared spectroscopy, multi-distance probe configuration, modified Beer–Lambert law with partial path length, activation map, classification

## Abstract

To improve the spatial resolution of imaging and get more effective brain function information, a multi-distance probe configuration with three distances (28.2, 40, and 44.7 mm) and 52 channels is designed. At the same time, a data conversion method of modified Beer–Lambert law (MBLL) with partial pathlength (PPL) is proposed. In the experiment, three kinds of tasks, grip of left hand, grip of right hand, and rest, are performed with eight healthy subjects. First, with a typical single-distance probe configuration (30 mm, 24 channels), the feasibility of the proposed MBLL with PPL is preliminarily validated. Further, the characteristic of the proposed method is evaluated with the multi-distance probe configuration. Compared with MBLL with differential pathlength factor (DPF), the proposed MBLL with PPL is able to acquire more obvious concentration change and can achieve higher classification accuracy of the three tasks. Then, with the proposed method, the performance of the multi-distance probe configuration is discussed. Results show that, compared with a single distance, the combination of the three distances has better spatial resolution and could explore more accurate brain activation information. Besides, the classification accuracy of the three tasks obtained with the combination of three distances is higher than that of any combination of two distances. Also, with the combination of the three distances, the two-class classification between different tasks is carried out. Both theory and experimental results demonstrate that, using multi-distance probe configuration and the MBLL with PPL method, the performance of brain function detected by NIRS can be improved.

## Introduction

Functional near-infrared spectroscopy (fNIRS) is a widely used non-invasive functional neuroimaging technology (Sato et al., 2016). Moreover, fNIRS is a safe, low-noise, portable, easy-to-use, and low-cost technique (Naseer and Hong, 2015). It has a higher spatial resolution than electroencephalogram and has a better temporal resolution than functional magnetic resonance imaging (fMRI). FNIRS device measures the intensity of light after passing through a certain brain area. Then the measured light intensity could be converted to the change of oxygenated hemoglobin (HbO) and deoxygenated hemoglobin (HbR) concentrations with some algorithms, and further the activation state of this area can be determined. Since first implemented about 25 years ago, several results have proven that fNIRS is an effective tool to study brain function and disease (Hoshi and Tamura, 1993; Kato et al., 1993; Villringer et al., 1993; Hoshi, 2005; Fujimoto et al., 2014). Now brain–computer interfaces based on fNIRS and the clinical application of fNIRS both have significant growths (Naseer and Hong, 2013; Khan et al., 2014, 2018; Naseer et al., 2014; Hong et al., 2015; Naseer and Hong, 2015).

For fNIRS, there are three typical techniques: continuous wave (CW), frequency domain, and time domain (Scholkmann et al., 2014). CW-fNIRS is one of the most popular fNIRS techniques. For a certain CW-fNIRS instrument, its temporal resolution is limited within a range and cannot be improved. However, the spatial resolution both in depth and laterally could be improved with some methods, for example, designing an appropriate probe configuration. Probe configuration refers to the distribution and connections between sources and detectors. A connection between a source and a detector is called a channel, and the distance of the channel is the separation of the source and the detector. In the early applications of fNIRS, the probe configurations of experiments were based on single-distance channel (Maki et al., 1995; Franceschini et al., 2003). Later, probe configuration with multi-distance channel was proposed. Once proposed, it was continuously adopted in the experiment. In 2014, Gagnon et al. (2014) used two kinds of multi-distance channels, eight channels of 10 mm and four channels of 30 mm, in a finger tipping task to improve the reduction of superficial noise. In 2016, Nguyen et al. proposed a bundled-optode method, which contains seven kinds of distances ranging from 25 to 50 mm, to detect the changes of HbO and HbR concentrations (Hoang-Dung and Hong, 2016). In 2017, adopting a probe configuration with multi-distance channels of 15, 21.2, 30, and 33.5 mm, Shin et al. (2017) verified the effectiveness of multi-distance channels to enhance the performance of fNIRS-BCI.

Besides, there are many algorithms to convert the measured light intensity to the concentration change of HbO and HbR, and the modified Beer–Lambert law (MBLL) is one of the most used algorithms (Scholkmann et al., 2014). As human tissue is a strong scattering medium for the near-infrared light, the Beer–Lambert law cannot be directly applied to biological tissue. British Delpy et al. took the light scattering into account and developed the MBLL in 1988 (Delpy et al., 1988). For MBLL, the differential pathlength factor (DPF) is usually estimated as a constant (about 6) (Duncan et al., 1995). However, for multi-distance probe configuration, light will penetrate different depths and pass through different brain tissue layers. For shorter distance channels, light may only pass through the scalp and skull (Strangman et al., 2014; Sato et al., 2016), whereas for longer distance channels, light is possible to pass through the cerebrospinal fluid (CSF), gray matter (GM), and white matter (WM) (Okada et al., 1997). Therefore, for multi-distance probe configuration, it will be more reasonable to take the pathlength of different layers into consideration. In the study of quantifying the influence of scalp and skull thickness on sensitivity of near-infrared neuromonitoring, Strangman et al. (2014) concluded the relationship between partial pathlength (PPL) of different layers and the channel length. However, they did not apply this relationship into the calculation of HbO and HbR concentration change.

To improve the spatial resolution of imaging and obtain more effective brain activation information, a multi-distance channel with 52 channels is designed, which includes 12 channels of 28.2 mm, 24 channels of 40 mm, and 16 channels of 44.7 mm. Besides, a method of MBLL with PPL is proposed to compute the concentration change of HbO and HbR. For a preliminary verification, another single-distance channel with 24 channels of 30 mm is also implemented. An experiment of three tasks, grip-stretch of left hand (LG) and right hand (RG) and rest (RE), is carried out with eight subjects. The reconstructed activation map and the classification accuracy are shown to reflect the performance of the proposed method, compared with MBLL with DPF. Then, with the proposed method, a classification is carried out to compare the performances of different distance combinations of the multi-distance probe configuration. Besides, with all 52 channels of the multi-distance probe configuration and MBLL with PPL, a classification of different tasks is also implemented.

## Materials and Methods

### fNIRS Instrument and Subjects

A three-wavelength (740, 808, and 850 nm) CW-fNIRS instrument (NirScan; Danyang Huichuang Medical Equipment, China) is utilized to acquire the fNIRS signals. There are 24 sources and 42 detectors in total, and more than 100 channels can be created by the combination of those sources and detectors. Emitted by all sources, the raw light intensity is a certain unknown constant. The remaining light intensity detected is stored by the instrument and then transported to a computer. The maximum sampling rate is set to 50 Hz, and the minimum dynamic range could reach 120 dB.

Eight subjects (five males and three females, ages range between 22 and 24) are invited to perform the experiment. All subjects are healthy and right handed. None of them have any neurological impairment or mental disorder. Before the experiments, the precautions of the experiment are clearly explained to the subjects. During the experiment, subjects are sitting in a comfortable chair in front of a computer. All experiments strictly adhere to the ethical standards and standard biosecurity and institutional safety procedures.

### Probe Configuration and Experiment Design

**FIGURE 1:**
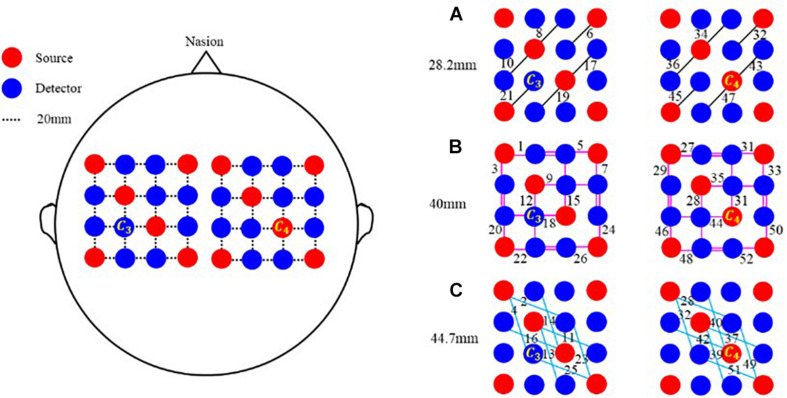
Multi-distance probe configuration. Red and blue circles represent sources and detectors, respectively. In the horizontal and vertical directions, the distance between two adjacent probes is 20 mm. Black line segments in **(A)** represent channels of 28.2 mm, pink segments in **(B)** represent channels of 40 mm, and sky-blue line segments in **(C)** represent channels of 44.7 mm.

To explore more active areas and improve the spatial resolution of imaging, a multi-distance probe configuration is designed. As shown in the left panel of Figure 1, this probe configuration is composed of 12 sources and 20 detectors, which are made up of two separate parts. Indicated with a black dotted line, the distance between two adjacent transducers is 20 mm along both horizontal and vertical directions. As depicted in the right panel, various pairings of sources and detectors make three kinds of channels with different distances. Marked with a black solid line, the shortest channel configures a 28.2 mm source–detector distance. Respectively identified with pink and sky-blue solid lines, the other two channels correspond to a 40 mm and a 44.7 mm source–detector distance. When the source–detector distances are 28.2, 40, and 44.7 mm, the corresponding numbers of channels are 12, 24, and 16. For this probe configuration, the total number of channels is 52. Also, the position of each channel is defined as the midpoint of the corresponding source and detector. For instance, if the positions of source and detector are (x_1_, y_1_, z_1_) and (x_2_, y_2_, z_2_), the coordinate of the channel is computed as (x, y, z) = [(x_1_ + x_2_)/2, (y_1_ + y_2_)/2, sqrt((x_1_ - x_2_)^2^ + (y_1_ - y_2_)^2^)/2].

**FIGURE 2:**
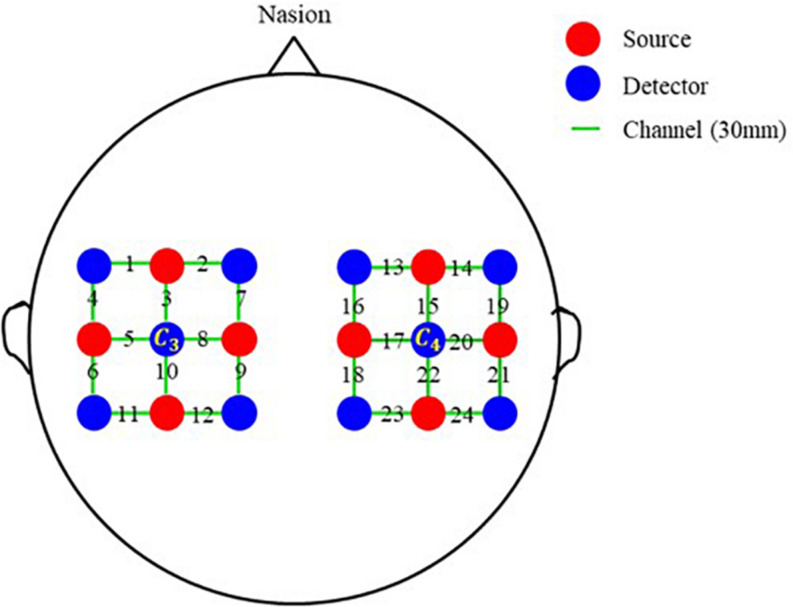
Single-distance probe configuration. Red and blue circles represent sources and detectors, respectively. In the horizontal and vertical directions, the distance between two adjacent probes is 30 mm. Green line segments represent channels, and the distance of all channels is 30 mm.

To make a preliminary verification, a single-distance probe configuration with 8 sources, 10 detectors, and 24 channels is also designed. As exhibited in Figure 2, the probe configuration is also made up of two separate parts. One is centered on *C*_*3*_ and covers the left-brain area; another is centered on *C*_*4*_ and covers the right-brain area. Each part contains 4 sources and 5 detectors, and 12 channels. The distance between two adjacent transducers is 30 mm in both horizontal and vertical directions, and the length of all channels is 30 mm.

**FIGURE 3:**
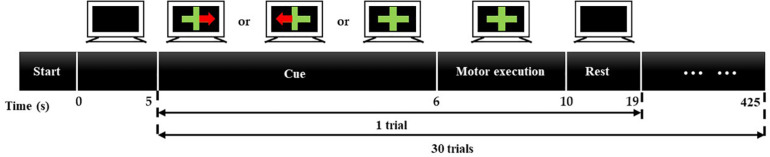
Experiment process of a block. A block will last for 425 s, including 5-s baseline acquisition and 30 trials. Every trial is 14 s, 1 s for cuing, 4 s for task, and 9 s for resting.

For this experiment, eight blocks are implemented, four for the single-distance probe configuration and four for multi-distance probe configuration. Figure 3 illustrates the process of a block. As can be seen, each block will last for 425 s, including 5 s baseline acquisition and 30 trials. Every trial is 14 s, 1 s for cuing, 4 s for task and 9 s for rest. The baseline signal is only acquired at the beginning of a block. When acquiring the baseline signal, the computer screen is black, and subjects sit in the chair in front of the computer with a relaxed state. During the cuing, three kinds of cues, a green cross with red-left arrow, a green cross with red-right arrow, or a green cross, will appear on the screen at random. When the cue appears, subjects should make a preparation for the corresponding task. The cue of green cross represents the task of rest (RE), green cross with red-left arrow represents the grip of left hand (LG), and green cross with red-right arrow represents the grip of right hand (RG). Later, a cross will appear on the screen and subjects will perform the corresponding task for 4 s according to the cue. During the task period, the grip of left hand or right hand will be performed four or five times. Then the green cross will disappear, and the computer screen turns black. At that moment, subjects could have a short rest for 9 s. In a block, each task will be executed 10 times. Therefore, for a probe configuration, each task would be executed 40 times.

### Concentration Convert

First, according to formula (1), the stored remaining light intensity is normalized. *i*, *t*, and λ represent the channel number, time, and wavelength, respectively. For *i*−th channel, *I*^i^(*t*, λ) and $I_{\text{out}}^{\text{i}}\,\left(t,\lambda \right)$ are the normalized and remaining light intensity at time *t* of wavelength λ, and $I_{\text{out}}^{\text{i}}\,\left(\lambda \right)$ is the mean light intensity of wavelength λ.


(1) $$I^{i}\left(t,\lambda \right)=I_{out}^{i}\left(t,\lambda \right)/I_{out}^{i}\left(\lambda \right)$$

For MBLL with DPF, the function between the normalized light intensity and concentration changes of HbO and HbR can be described as formulas (2) and (3). Δ*OD*, a unitless value, represents the change of optical density. ε is the molar extinction coefficients, and the values of different wavelengths are listed in Table 1 (Matcher et al., 1995). Δ*C* is the concentration change. *d* is the length of a channel.

**TABLE 1:** Extinction coefficient at wavelengths of 740, 808, and 850 nm.

λ (nm)	ε_*HbO*_(*mM*^−1^*cm*^−1^)	ε_*HbR*_(*mM*^−1^*cm*^−1^)
740	0.4920	1.3411
808	0.8164	0.8040
850	1.1596	0.7861


(2) $$\Delta OD^{i}\left(t,\lambda \right)=-\log_{10}\left[I^{i}\left(t,\lambda \right)\right]=[\varepsilon_{HbO}\left(\lambda \right)\Delta C_{HbO}^{i}\left(t\right)+\varepsilon_{HbR}\left(\lambda \right)\Delta C_{HbR}^{i}\left(t\right)]DPF\cdot d$$

Taking three wavelengths into consideration, Δ*C* could be calculated with formula (3). For MBLL with DPF, the value of *DPF* is a constant and usually takes 6. The pathlength is the product of *DPF* and *d*, through which light passes. However, for multi-distance probe configuration, light will penetrate different depths and pass through different brain tissue layers, and the pathlength is different for different probe distances.


(3)  [ΔCHbOi(t)ΔCHbRi(t)]=d-1⋅A⋅-1B,A=[εHbO(λ1)εHbO(λ2)εHbO(λ3)εHbR(λ1)εHbR(λ2)εHbR(λ3)],B=[ΔODi(t,λ1)/DPFΔODi(t,λ2)/DPFΔODi(t,λ3)/DPF]

Taking the PPL of different layers into consideration, an improved method, MBLL with PPL, is proposed and investigated. First, the relationships between the PPL of different layers and channel length are expressed by formula (4)–(8) based on the simulation data in Strangman et al. (2014). The pathlength (PL) can be computed by formula (9), where *PPL*^*scalp*^, *PPL*^*skull*^, *PPL*^*CSF*^, *PPL*^*GM*^, and *PPL*^*WM*^ are the partial pathlength of scalp, skull, CSF, GM, and WM, respectively.


(4) $$PPL^{scalp}=\left\{ \begin{array}{c} 0.75\times d+37.5,20\leq d\leq 35\\ 0.4\times d+48.5,35\leq d\leq 60 \end{array}\right.$$

(5) PPLskull={2.71×d-1,  20≤d≤35 1.2×d+33, 35≤d≤60 

(6) PPLCSF={d-15,20≤d≤45 1.17×d-22.5,45≤d≤60

(7) PPLGM={d-15,20≤d≤45 0.92×d-11.2.5,45≤d≤60

(8) $$PPL^{WM}=0.1\times d-2,20\leq d\leq 60$$

(9) $$PL=PPL^{scalp}+PPL^{skull}+PPL^{CSF}+PPL^{GW}+PPL^{MW}$$

When taking PPL into consideration, formula (2), (3) would be transformed into formula (10), (11). For channels of different lengths, PL can be calculated according formula (4)–(9), and then the value of PL is used in formula (11) to get the concentration changes of HbO and HbR.


(10) $$\Delta OD^{i}\left(t,\lambda \right)=-\log_{10}\left[I^{i}\left(t,\lambda \right)\right]=[\varepsilon_{HbO}\left(\lambda \right)\Delta C_{HbO}^{i}\left(t\right)+\varepsilon_{HbR}\left(\lambda \right)\Delta C_{HbR}^{i}\left(t\right)]PL$$

(11) $$\left[\begin{array}{c} \Delta C_{HbO}^{i}\left(t\right)\\ \Delta C_{HbR}^{i}\left(t\right) \end{array}\right]=A^{-1}\cdot C,A=\left[\begin{array}{cc} \begin{array}{c} \varepsilon_{HbO}\left(\lambda_{1}\right)\\ \varepsilon_{HbO}\left(\lambda_{2}\right)\\ \varepsilon_{HbO}\left(\lambda_{3}\right) \end{array}\begin{array}{c} \varepsilon_{HbR}\left(\lambda_{1}\right)\\ \varepsilon_{HbR}\left(\lambda_{2}\right)\\ \varepsilon_{HbR}\left(\lambda_{3}\right) \end{array} & \end{array}\right],C=\left[\begin{array}{c} \Delta OD^{i}\left(t,\lambda_{1}\right)/PL\\ \Delta OD^{i}\left(t,\lambda_{2}\right)/PL\\ \Delta OD^{i}\left(t,\lambda_{3}\right)/PL \end{array}\right]$$

For each block, the recorded light intensity is first translated into the change of optical density. Second, the data are bandpass filtered between 0.01 and 0.2 Hz. Third, MBLL with PPL and MBLL with DPF are adopted, respectively, to accomplish the concentration convert. Then, trials are extracted and the concentration change within the 0–5 s of a block is considered as the baseline and subtracted from each trial in this block.

### Classification

To verify the advantages of the proposed method over the MBLL with DPF, classification of the three tasks is performed with the MBLL with DPF and the proposed method, respectively. Besides, with the proposed method and the multi-distance probe configuration, the classification is implemented to test the performance of the combination of different distances. For classification, support vector machine (SVM) is selected compared with linear discriminant analysis (LDA) and artificial neural networks (ANNs) because SVM is more suitable for ternary classifications than LDA and is easier than ANN in operation. The template of SVM is created by the Matlab function *templateSVM*. In the template, “Standardize” is set to 1 and others are set as default options. Second, five feature vectors are constructed, including mean, variance, maximum, skewness, and kurtosis of concentration change of HbO. The calculation method of the five features refers to the paper of Naseer et al. (2016). Considering the effect of hemodynamic delay, features are calculated with the concentration change of HbO in the time window of 2–8 s for every trial. The classification of different tasks is also discussed, including the binary classification (LG/RG, LG/RE, RG/RE) and ternary classification (LG/RG/RE).

To make classification accuracy more reliable, a 10-fold cross-validation is carried out 10 times for every subject. Besides, to make the results more convincing, the average of the 10 times’ accuracies is calculated as the final accuracy for every subject.

## Results

### Timing Analysis of Measurement Data

**FIGURE 4:**
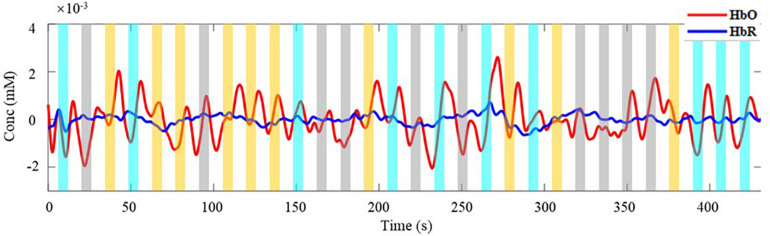
The average concentration changes of HbO and HbR. Red line is the change of HbO, blue line is the change of HbR. Rectangles of different colors represent different task. The yellow, cyan-blue, and gray represent the task of LG, RG, and RE, respectively.

For the experiment, it is critical to ensure the effectiveness of the measured data. With MBLL, the averaged concentration changes of all channels are analyzed first. Taking single-distance probe configuration for example, Figure 4 is the concentration changes of HbO and HbR of a subject within a block. The time window covered by the rectangle is the 1–7 s of each trial (the time distribution of a trial is shown in Figure 3), including the task period and the 2 s immediately after the end of the task period. The rectangles of different colors represent different tasks. The yellow, cyan-blue, and gray represent the task of LG, RG, and RE, respectively. As can be seen, the change of HbO is more significant than HbR, which is consistent with the human physiological changes. Also, the concentration change of HbO is discussed in the following analysis.

**FIGURE 5:**
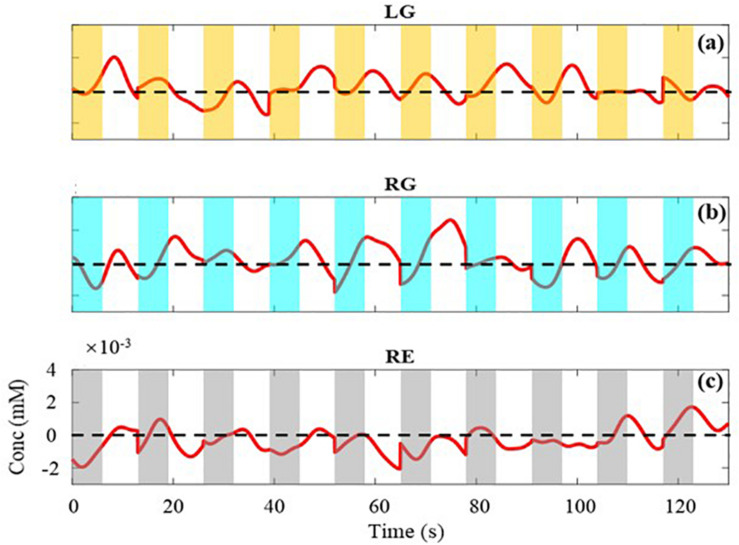
The average concentration changes of HbO for each task. **(a–c)** are the changes of LG, RG, and RE, respectively. The value of the black horizontal dotted line is 0.

As illustrated in Figure 4, three kinds of tasks are performed at random and each task is performed 10 times in a block. The concentration changes of HbO of each task in Figure 4 are extracted and displayed in Figure 5. Figures 5a–c, respectively, represent the task of LG, RG, and RE. The value of the black horizontal dotted line is 0, which means the concentration has not changed. Below and above the black line indicate a relative decrease and increase in concentration, respectively. As the trial in the blue dotted frame, during trials, the concentration changes of HbO both have a tendency of rising to the maximum and then falling. At the beginning and end of a trial, the values of the concentration are almost less than 0. Also, because of the delay of hemodynamic response, the maximum comes after several seconds of the task period of a trial (Gagnon et al., 2011; Buxton, 2012). For the task of LG and RG, the range of the concentration change is bigger than that of RE. Besides, for LG and RG, the values of the concentration are positive numbers for most of the time, whereas for the task of RE, the values of the concentration changes are negative numbers for most of the time. This phenomenon indicates that the movements of left and right hands could cause obvious increases in the concentration of HbO and confirms the effectiveness of the measured data.

### Performance of MBLL With PPL Compared With MBLL With DPF

**FIGURE 6:**
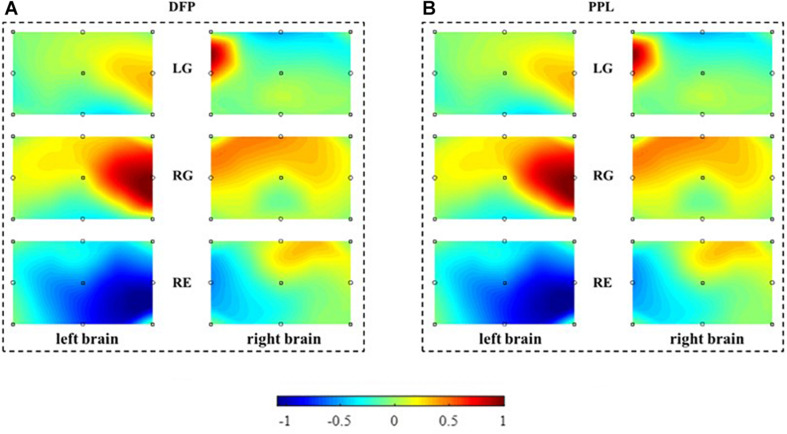
The reconstructed brain activation maps. The marks in the panels represent the sources and detectors. The color bar, denoting the activation strength, is normalized according to the values of concentration change of HbO. **(A)** Reconstructed brain activation maps with MBLL with DPF. **(B)** Reconstructed brain activation maps with MBLL with PPL.

To evaluate the performance of the MBLL with PPL, a brain activation map is reconstructed and compared first. With the single-distance probe configuration, Figure 6 is the brain activation maps reconstructed from the calculated concentration changes of HbO. Figure 6A is constructed from the method of MBLL with DPF, and (B) is constructed from the proposed method, MBLL with PPL. For (A) and (B), the left column is the brain activation map of the left brain and the right column is the brain activation map of the right brain. The first, second, and third rows represent the task of LG, RG, and RE, respectively. In Figure 6B, there is a relatively more active area in the right brain for the task of LG. Similarly, there is a relatively more active area in the left brain for the task of RG. This phenomenon is consistent with the characteristics of contralateral dominance of the brain (Cernacek, 1961). For the task of RE, the average concentration of HbO (Figure 5C) is less than 0, and correspondingly, there are no active areas in the left and right brain. As shown in Figure 6A, for MBLL with DPF, the brain activation map has the same characteristic, and the corresponding active areas are almost the same as that in Figure 6B. The good consistency demonstrates the correctness of the proposed method.

**FIGURE 7:**
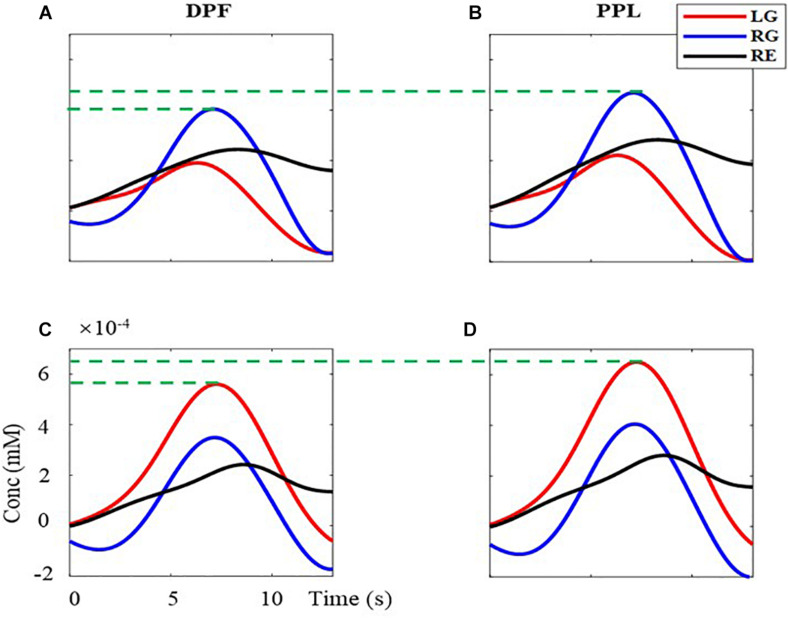
The superimposed average concentration changes of HbO with the single-distance probe configuration. The red, blue, and black solid line are the signal of LG, RG, and RE, respectively. **(A,B)** are the concentration changes of left-brain, **(C,D)** are the concentration changes of right-brain. **(A,C)** are calculated with MBLL with DPF, **(B,D)** are calculated with MBLL with PPL.

Besides, the 40 trials’ signals of HbO are superimposed averaged for each task. First, the baseline is subtracted for every trial. Second, the signal in the time windows of 1–14 s (task period and rest period of a trial) are extracted and superimposed averaged. Third, the obtained 13-s singles in the time window are presented in Figure 7. The left column (Figures 7A,C) is the results calculated by the MBLL with DPF, the right column (Figures 7B,D) is the results calculated by the MBLL with PPL. The first row (Figures 7A,B) and second row (Figures 7C,D) are the signal of the left brain and right brain, respectively. As can be seen, all signals have an upward to the maximum and then downward trend. For the signal of the left brain (Figures 7A,B), the range and maximum of the task of RG are both larger than tasks of LG and RE. Similarly, for the signal of the right brain (Figures 7C,D), the range and maximum of the task of LG are both larger than tasks of RG and RE. This phenomenon reflects the characteristics of contralateral dominance of the brain (Cernacek, 1961). As indicated by the green dotted line in Figures 7A,B, for the task of RG, the maximum acquired by MBLL with PPL is 16.06% bigger than that acquired by MBLL with DPF. Also, for the task of LG in Figures 7C,D, the maximum acquired by MBLL with PPL is also 16.06% bigger than that acquired by MBLL with DPF. That is, for the same measured data, the proposed method is able to acquire more obvious concentration change. Also, the more obvious concentration change on brain function change is very beneficial in clinical application and fNIRS-BCI.

**FIGURE 8:**
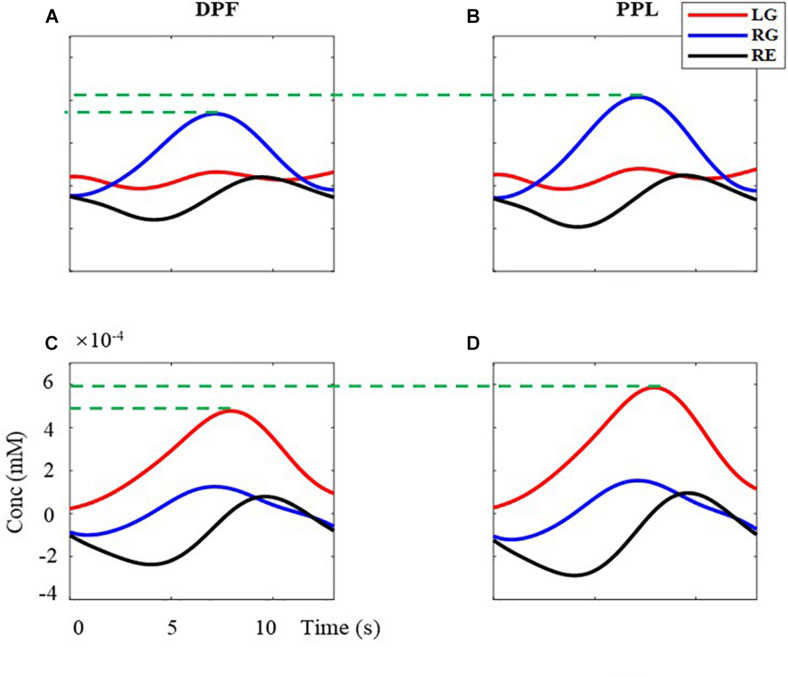
The superimposed average concentration changes of HbO with the multi-distance probe configuration. The red, blue, and black solid line are the signal of LG, RG, and RE, respectively. **(A,B)** are the concentration changes of left-brain, **(C,D)** are the concentration changes of right-brain. **(A,C)** are calculated with MBLL with DPF, **(B,D)** are calculated with MBLL with PPL.

What is more, the effectiveness of the proposed MBLL with PPL is analyzed with the multi-distance probe configuration. Figure 8 is the superimposed averaged signals. The left column (Figures 8A,C) is the results computed by the MBLL with DPF, the right column (Figures 8B,D) is the results computed by the proposed method. The first row (Figures 8A,B) and second row (Figures 8C,B) are the signal of left brain and right brain, respectively. For the task of RG, the maximum value of (B) is 23.20% bigger than that of (A). For the task of LG, the maximum value of (D) is 23.15% bigger than that of (C). The effectiveness of the proposed MBLL with PPL is further verified with multi-distance probe configuration.

In addition to the comparison of the activation map and the waveform, based on the multi-distance probe configuration, classifications of the three tasks are performed with both methods, MBLL with DPF and MBLL with PPL. For every subject, a 10-fold cross-validation is carried out 10 times. The result for every subject is the average of the 10 times’ accuracies. As exhibited in Table 2, the proposed method could achieve higher accuracy for every subject. Based on the MBLL with PPL, the average accuracy of all subjects is 57.33%, which is 5.92% higher than that of MBLL with DPF (51.41%). Besides, the result of the Wilcoxon signed-rank test, a non-parametric statistics test method, shows a significant difference of the accuracy rate between MBLL with PPL and MBLL with DPF (*p* = 0.012) for the eight subjects.

**TABLE 2:** The three tasks’ classification accuracies (%) of the method of MBLL with DPF and MBLL with PPL (the letter “S” represents “subject”).

	S1	S2	S3	S4	S5	S6	S7	S8	Mean
DPF	43.92	86.75	38.50	33.50	41.08	57.17	51.25	59.08	51.41
PPL	45.08	94.08	46.92	36.92	44.50	61.08	62.67	67.42	57.33

### Discussion on Different Distance Combinations

**FIGURE 9:**
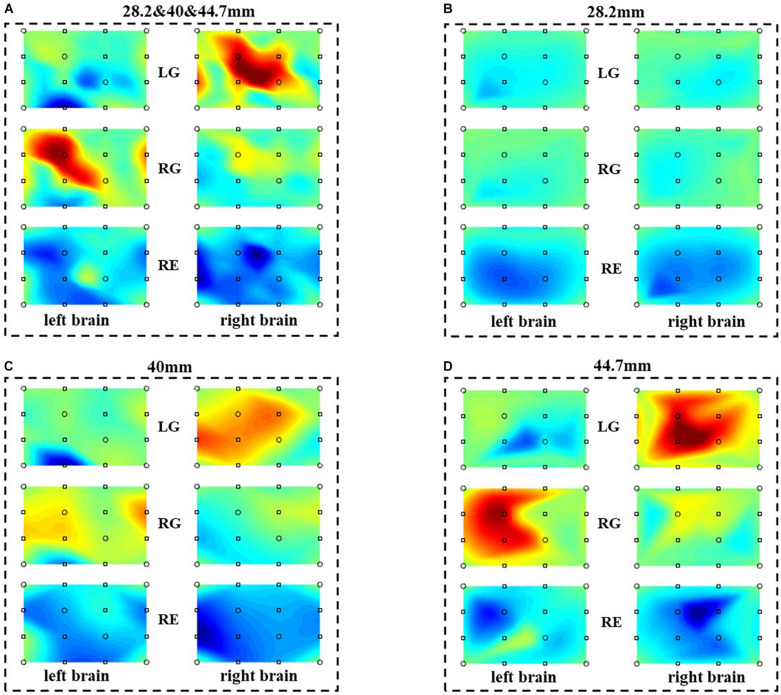
The reconstructed brain activation maps with the multi-distance probe configuration. The marks in the figure represent the sources and detectors. **(A–D)** are the reconstructed brain activation maps with the combination of 28.2 and 40 and 44.7 mm, 28.2 mm, 40 mm, 44.7 mm respectively.

With the proposed method, the performance of the combinations of different distance is discussed. Figure 9 is the reconstructed brain activation maps of different distance combinations. Figure 9A is the activation map of the multi-distance probe configuration, with three distances (28.2, 40, 44.7 mm). Figures 9B–D are the activation maps of single distance probes, with 28.2, 40, and 44.7 mm, respectively. As illustrated in Figures 9A,C,D, for the task of LG and RG, the results are consistent with the characteristics of contralateral dominance of the brain (Cernacek, 1961). What is more, the relative active areas in (C) and (D) also exist in (A), and the corresponding areas in (A) are more accurate than those of (B) and (D). Compared with the single distance probe configuration, the multi-distance probe configuration has a better spatial resolution and could explore more accurate information. For the task of RE, there is no active area both in the left and right brain.

**FIGURE 10:**
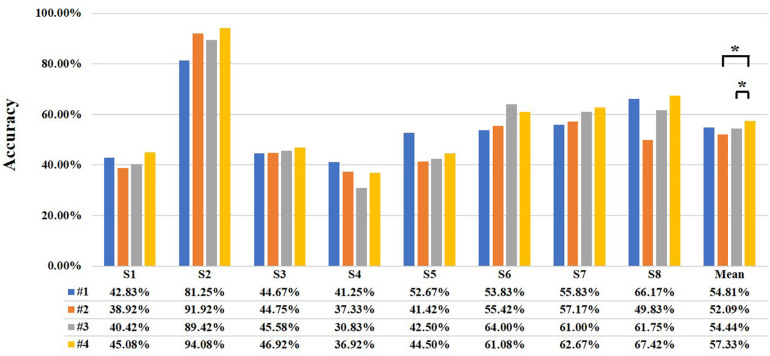
The classification accuracy of the different distance combinations (* represents significant difference, *p < 0.05 and **p < 0.01). The four different colors represent different combinations and are listed at the bottom. The #1, #2, #3, and #4 represent the combination of 28.2 and 40 mm, 28.2 and 44.7 mm, 40 and 44.7 mm, and 28.2 and 40 mm and 44.7 mm, respectively. The letter “S” represents “subject.”

Then, with MBLL with PPL and multi-distance probe configuration, a three-class classification is carried out to test the performance of the different distance combinations. Four kinds of distance combinations are discussed, including 28.2 and 40 mm (#1), 28.2 and 44.7 mm (#2), 40 and 44.7 mm (#3), and 28.2 mm and 40 mm and 44.7 mm (#4). Figure 10 shows the classification accuracies of the four combinations of every subject. For most subjects, the performance of the combination of 28.2 + 40 + 44.7 mm is the best, the maximum accuracy of a subject is up to 94.08%, and the mean of all subjects is 57.33%. For the combination of 28.2 + 40 mm, 28.2 + 44.7 mm, and 40 + 44.7 mm, the highest accuracies of a subject are 81.25, 91.92, and 89.42%, respectively. The mean accuracies are 54.81, 52.09, and 54.44%. For different combinations, the mean accuracy of #4 is 2.52, 5.24, and 2.89% higher than that of #1, #2, and #3. Besides, Wilcoxon signed-rank test yields a significant difference of classification accuracy between #2 and #4, and #3 and #4.

Further, classification of the combination of different tasks is performed with all channels in the multi-distance probe configuration. The classification accuracies are presented in Table 3. As displayed, in the binary classification, the mean accuracy of the LG/RG (73.30%) is the highest, and the mean accuracy of LG/RE (69.69%) and RG/RE (65.00%) are slightly lower. One of the reasons is that, compared with nothing to do, the subjects are more focused when performing movements. The mean accuracy of the ternary classification (57.33%) is less than all binary classifications. The highest accuracies are 97.88, 97.88, 88.88, and 94.08% for the four different task combinations. Like the aforementioned, the value of the final accuracy is the average of the 10 times’ accuracies for every subject.

**TABLE 3:** The classification accuracy of the different task combinations.

Subject	Classification accuracy (%)			
	LG/RE	LG/RG	RG/RE	LG/RG/RE
S1	72.63	57.00	47.88	45.08
S2	97.88	97.88	88.88	94.08
S3	72.25	54.38	66.63	46.92
S4	51.13	58.50	49.38	36.92
S5	50.50	66.38	55.63	44.50
S6	75.25	79.50	63.38	61.08
S7	70.25	84.50	69.63	62.67
S8	67.63	88.25	78.63	67.42
Mean	69.69	73.30	65.00	57.33

## Conclusion and Discussion

In this study, a multi-distance probe configuration with three distances (28.2, 40, and 44.7 mm) is designed, and the data conversion method of MBLL with PPL is proposed. With a single-distance probe configuration, the feasibility of the proposed method is validated first. Compared with MBLL with DPF, the proposed MBLL with PPL is of good performance, which is able to acquire larger brain function change and could acquire higher classification accuracy. Besides, the activation map of multi-distance probe configuration contains more accurate brain function information. In addition, when all channels are applied, the designed multi-distance probe has the best classification performance, and the mean classification accuracy is 2.52, 5.24, and 2.89% higher than the other distance combinations. Finally, the classification of different tasks is also discussed.

All results indicate the work’s potential for detecting higher spatial resolution brain function. For the reconstructed brain activation map of 28.2 mm in Figure 9B, the characteristics of the opposite side of the brain are not reflected. This may be caused by the joint of low-density measurement and shorter distance of channel. For the multi-distance probe configuration, the combination of the three distances has the best performance than other combinations, and it is likely to acquire better performance with significant differences by optimizing the experimental paradigm. Several works have revealed that the shorter distance in a multi-distance probe configuration could be used to remove the surficial noise (Lee et al., 2002; Scremin and Kenney, 2004; Bauer et al., 2006; Gagnon et al., 2011; Saager et al., 2011). In the following studies, the shorter distance will be taken into consideration for removing the surficial noise and more work will be done to explore the experimental paradigm.

## Data Availability Statement

The raw data supporting the conclusions of this article will be made available by the authors, without undue reservation, to any qualified researcher.

## Ethics Statement

XC conceived, designed and performed the experiment, and wrote the manuscript. XS, LC, and XA supervised the experiment. XC and XS analyzed the data and are responsible for data curation. XS revised the manuscript. XS and DM were responsible for project administration. DM provided resources. All authors approved the final manuscript.

## Author Contributions

XC conceived, designed and performed the experiment, and wrote the manuscript. XS, LC, and XA supervised the experiment. XC and XS analyzed the data and are responsible for data curation. XS revised the manuscript. XS and DM were responsible for project administration. DM provided resources. All authors approved the final manuscript.

## Conflict of Interest

The authors declare that the research was conducted in the absence of any commercial or financial relationships that could be construed as a potential conflict of interest.
