# Corrigendum: Performance Improvement for Detecting Brain Function Using fNIRS: A Multi-Distance Probe Configuration With PPL Method

**DOI:** 10.3389/fnhum.2021.786352

**Published:** 2021-11-04

**Authors:** Xinrui Chen, Xizi Song, Long Chen, Xingwei An, Dong Ming

**Affiliations:** ^1^Academy of Medical Engineering and Translation Medicine, Tianjin University, Tianjin, China; ^2^College of Precision Instruments and Optoelectronics Engineering, Tianjin University, Tianjin, China

**Keywords:** functional near-infrared spectroscopy, multi-distance probe configuration, modified Beer-Lambert law with partial path length, activation map, classification

Xinrui Chen was not included as a first author in the published article. Xinrui Chen and Xizi Song now share first authorship. The corrected Author Contributions Statement appears below.

## Author Contributions

XC conceived, designed and performed the experiment, and wrote the manuscript. XS, LC, and XA supervised the experiment. XC and XS analyzed the data and are responsible for data curation. XS revised the manuscript. XS and DM were responsible for project administration. DM provided resources. All authors approved the final manuscript.

The authors apologize for this error and state that this does not change the scientific conclusions of the article in any way. The original article has been updated.

## Publisher's Note

All claims expressed in this article are solely those of the authors and do not necessarily represent those of their affiliated organizations, or those of the publisher, the editors and the reviewers. Any product that may be evaluated in this article, or claim that may be made by its manufacturer, is not guaranteed or endorsed by the publisher.

